# *ndufa7* plays a critical role in cardiac hypertrophy

**DOI:** 10.1111/jcmm.15921

**Published:** 2020-09-29

**Authors:** Xingjuan Shi, Yu Zhang, Ru Chen, Yijie Gong, Mingming Zhang, Rui Guan, Ori D. Rotstein, Xiangdong Liu, Xiao‐Yan Wen

**Affiliations:** ^1^ School of Life Science and Technology Key Laboratory of Developmental Genes and Human Disease Southeast University Nanjing China; ^2^ Zebrafish Centre for Advanced Drug Discovery Keenan Research Centre for Biomedical Science Li Ka Shing Knowledge Institute St. Michael’s Hospital Toronto Ontario Canada; ^3^ Department of Medicine & Institute of Medical Science University of Toronto Toronto Ontario Canada

**Keywords:** cardiac hypertrophy, *ndufa7*, nppa, nppb, zebrafish

## Abstract

Cardiac hypertrophy is a common pathological change in patients with progressive cardiac function failure, which can be caused by hypertrophic cardiomyopathy (HCM), dilated cardiomyopathy (DCM) or arterial hypertension. Despite years of study, there is still limited knowledge about the underlying molecular mechanisms for cardiac hypertrophy. NDUFA7, a subunit of NADH:ubiquinone oxidoreductase (complex I), has been reported to be a novel HCM associated gene. However, the biological role of NDUFA7 in heart remains unknown. In this study, we found that NDUFA7 exhibited high expression in the heart, and its level was significantly decreased in mice model of cardiac hypertrophy. Moreover, we demonstrated that *ndufa7* knockdown in developing zebrafish embryos resulted in cardiac development and functional defects, associated with increased expression of pathological hypertrophy biomarkers nppa (ANP) and nppb (BNP). Mechanistic study demonstrated that *ndufa7* depletion promoted ROS production and calcineurin signalling activation. Moreover, NDUFA7 depletion contributed to cardiac cell hypertrophy. Together, these results report for the first time that *ndufa7* is implicated in pathological cardiac hypertrophy.

## 1 INTRODUCTION

Cardiac hypertrophy is an adaptive response of the heart to haemodynamic and neurohormonal stress to preserve cardiac function.^1^ Prolonged cardiac hypertrophy can contribute to functional decompensation, cardiac fibrosis, even progress to heart failure or sudden death, leading to a high mortality worldwide.^2^ Cardiac hypertrophy is accompanied by reactivation of a set of cardiac foetal genes, including atrial natriuretic peptide (ANP, nppa), brain natriuretic peptide (BNP, nppb) and β‐myosin heavy chain (β‐MHC), as well as an increase in cell size and protein synthesis, suggesting that molecular events controlling heart development are redeployed to regulate hypertrophic growth.^3^ Among many intracellular signalling pathways involved in hypertrophic development, calcineurin/ nuclear factor of activated T cell (NFAT) pathway plays a key role in pathological cardiac hypertrophy.^4^ NFAT, resides in the cytoplasm in a hyper‐phosphorylated state, is dephosphorylated by the calcium/calmodulin‐activated phosphatase calcineurin and then translocate to the nucleus where it regulates transcription of hypertrophic genes.^5^

The heart is the greatest oxygen‐consuming organ in the body, which requires ATP production from mitochondrial respiration to maintain normal cardiac mechanical function.^6^ Therefore, mitochondrial dysfunction plays a key role in the pathogenesis and development of cardiac hypertrophy.^7^ Reactive oxygen species (ROS) are a natural by‐product of mitochondrial energy production.^8^ ROS are generated during excessive oxidative stress, which has been implicated in the pathogenesis of cardiac hypertrophy and heart failure.^9^ Interestingly, it has been reported that calcineurin regulates the pathogenesis of cardiac hypertrophy with accompanying by the intracellular ROS production.^10^ It has been demonstrated that ROS stimulates the signal transduction in cardiomyocytes during pathological conditions by activating NFAT.^11^

*NDUFA7*, also known as *B14.5*, encodes a subunit of NADH:ubiquinone oxidoreductase (complex I) in the mitochondrial respiratory chain. It has been reported that an association is observed between *NDUFA7* and rheumatoid arthritis (RA) with severe erosive arthritis.^12^ Our previous work of whole exome sequencing analysis on hypertrophic cardiomyopathy patients found that *NDUFA7* might be a novel candidate gene associated with cardiomyopathy.^13^ However, the biological role of *NDUFA7* in cardiac diseases has not been explored. Here we present the first evidence to our knowledge that *ndufa7* depletion contributes to pathological cardiac hypertrophy.

## 2 MATERIALS AND METHODS

### 2.1 Tissue expression profiles and animal model data

The Genotype‐Tissue Expression database (GTEx, http://www.gtexportal.org/) was used to investigate the expression of candidate genes in multiple human tissues. The zebrafish information network (ZFIN, http://zfin.org/) and the mouse genome database (MGD, http://www.informatics.jax.org/) databases, as well as PubMed, were used to investigate the phenotype of candidate genes in zebrafish and mice. Clustal Omega software (https://www.ebi.ac.uk/Tools/msa/clustalo/) was used for multiple sequence alignment.

### 2.2 Zebrafish care and breeding

All procedures were performed in accordance with the guidelines of the Canadian Council on Animal Care and approved by the Institutional Animal Care Committee. Wild‐type zebrafish embryos were used for recording heart rates. A transgenic *cmlc2::GFP* zebrafish line that marks cardiomyocytes with Green Fluorescence Protein (GFP) was used for heart morphology imaging studies or extraction for quantitative RT‐PCR. A transgenic *nppb::F‐luc* zebrafish line that expresses firefly luciferase under the control of the *nppb* promoter was employed as in vivo model labelling BNP pathway, a biomarker of heart failure. Wild‐type zebrafish was used for all other experiments. The zebrafish lines were maintained at 28℃ in 14:10 h light/dark conditions. Protocols for experimental procedures were approved by the Research Ethics Board of St. Michael's Hospital (Toronto) (protocol ACC660). Zebrafish were killed by immersion in an ice‐water (4°C or less) bath followed by overdose of clove oil (70%‐95% eugenol) or tricaine methanesulphonate (MS‐222, 200‐300 mg/L). All animal experiments were performed in accordance with the NIH guidelines on the protection of animals used for scientific purposes.

### 2.3 Morpholinos and expression constructs

Morpholino (MO)‐modified antisense oligonucleotides (Gene Tools) were designed against the splice donor site of zebrafish *ndufa7* exon 2 (*ndufa7* MO: 5’‐TCCGTTTCTTAACAGCAAGATCTCC‐3’). The morpholinos were injected into zebrafish embryos at the 1‐cell stage. As a negative control, a standard control oligonucleotide (Control MO: 5’‐CCTCTTACCTCAGTTACAATTTATA‐3’) was injected at the same dose.

### 2.4 RNA isolation, reverse transcription (RT‐PCR) and quantitative real‐time PCR (qPCR)

Total RNA was isolated from whole embryos, larval hearts, cells or tissues with Trizol reagent (Invitrogen) and reverse‐transcribed into cDNA with an Oligo dT primer and SuperScript II reverse transcriptase (Invitrogen). The efficiency of the *ndufa7* splice MO was tested by carrying out RT‐PCR from RNA extracted from zebrafish whole embryos at 2dpf with *ndufa7* primers. For the analysis of specific gene expression in zebrafish larval hearts or cells, real‐time PCR was performed on all samples in triplicate with a Power SYBR Green PCR Master Mix (Applied Biosystems) as described previously.^14^, ^15^ The 2^－△△Ct^ method was used to normalize the gene of interest to the endogenous housekeeping gene *rpl13a* (ribosomal protein L13a) or GAPDH and determine the fold change relative to control. Primers used for experiments were listed in Table 1.

**Table 1:** Primers for qPCR or ISH analysis

Use	Gene	Forward primer (5’→3’)	Reverse primer (5’→3’)
qPCR	ndufa7	ATCCAGAGGCTGAGGAATTATCTGTCAG	TCATGTCTTCACAGAAAGCTCTGACAGC
	nppa	GATGTACAAGCGCACACGTT	TCTGATGCCTCTTCTGTTGC
	nppb	CATGGGTGTTTTAAAGTTTCTCC	CTTCAATATTTGCCGCCTTTAC
	rpl13a	TCTGGAGGACTGTAAGAGGTATGC	AGACGCACAATCTTGAGAGCAG
	vmhc	TCAGATGGCAGAGTTTGGAG	GCTTCCTTTACAGTTACAGTCTTTC
	cmlc2	GTGATGAAGAGCTGGAGT	GGGTCATTAGCAGCCT
	serca	GGATCATCAACATCGGCCAC	CGTTCTTCTTGGCCATACGG
	calcineurin	GCCTTTAGGATCTACGACATGG	ATATTCTCCCGTCTCCGTCTTT
	NDUFA7	TACTGTACTCGTGATGGCCG	GACAGCTCCCACCTCTTCAT
	ANP	GAGGAGAAGATGCCGGTAG	CAGAGAGGGAGCTAAGTG
	BNP	TGATTCTGCTCCTGCTTTTC	GTGGATTGTTCTGGAGACTG
	GAPDH	ACAGCAACAGGGTGGTGGAC	TTTGAGGGTGCAGCGAACTT
ISH	ndufa7	TAATACGACTCACTATAGGGA AAAGAGAAAATCCAGAGGCTGAGGAATTATCTGTCAG	ATTAACCCTCACTAAAGGAAAAGGAGGGCTGTCAGAGCTTTCTGTGAAGACATGA
	nppa	TAATACGACTCACTATAGGGAAAAGAGAAAGGCAACAGAAGAGGCATCAG	ATTAACCCTCACTAAAGGAAAAGGAGGGGAAGACCCTATGCGATCCA
	nppb	TAATACGACTCACTATAGGGA AAAGAGAAAACCGGCGGAAAGAGAAGTAA	ATTAACCCTCACTAAAGGAAAAGGAGGCCCGACTGTGTTACATCCCA

### 2.5 Whole‐mount in situ hybridization (ISH)

For all manipulations, embryos at the appropriate developmental stage, 1 cell, 24 hpf (hour post‐fertilization), 48 hpf, 72 hpf and 96 hpf were fixed in 4% paraformaldehyde/PBS. In situ hybridization and immunohistochemistry were performed using standard protocols. Digoxigenin‐labelled riboprobes were prepared from PCR templates with T3 and T7 polymerases as recommended by the supplier (Roche). Staining was visualized using an anti‐DIG‐alkaline phosphatase‐conjugated antibody and NBT/BCIP (Roche). After probe detection, embryos were cleared and photographed in glycerol.

### 2.6 Cell culture and transfection

H9c2 cells, obtained from the Type Culture Collection of the Chinese Academy of Sciences (Shanghai, China), were cultured in the Dulbecco's modified Eagle's medium and maintained at 37℃ in a 5% CO_2_ humidified atmosphere. Rat NDUFA7 siRNA oligonucleotides (siNDUFA7#1: CAACAATTACTACTGTACT, siNDUFA7#2: ATCATCATGTCCTCACAAA) were synthesized (RiboBio) and transfected into cells with Lipofectamine RNAiMAX transfection reagent (Thermo Fisher Scientific) according to manufacturers’ protocols.

### 2.7 Drug exposures

FK506 (Sigma) were stored in DMSO stocks and diluted into working concentrations (1 μg/ml). The final DMSO concentration was 0.5% for all experiments. Embryos were manually dechorionated at 21 hpf, and FK506 was then applied. 2’,7’‐dichlorofluorescin diacetate (Sigma) was used as an indicator of ROS level. Embryos were stained with 2’,7’‐dichlorofluorescin diacetate at room temperature for an hour at 72 hpf and then imaged under the fluorescence microscope. The intensity of ROS level was examined using ImageJ software (National Institution of Health). For cellular ROS measurement, H9c2 cells were incubated with 2’,7’‐dichlorofluorescin diacetate at 37°C for 30 minutes, washed with PBS for three times and then visualized under the fluorescence microscope.

### 2.8 Luciferase assay

The *nppb::F‐Luc* embryos injected with morpholinos or treated with chemicals were placed into a 96‐well microtiter plate with 100 μL buffered embryo water and incubated at 28°C for embryonic development. The plate was removed from the incubator at 72 hpf, and 25 μL of long half‐life firefly luciferase reagent (Perkin Elmer, Steady‐Glo) was added to the well. After incubation for 1 hour in dark, the plate was read in a high‐sensitivity luminescence plate reader (SpetraMax M5e, Molecular Devices).

### 2.9 Immunoblotting

Protein was extracted in lysis buffer (50 mmol/L Tris, pH 7.5, 150 mmol/L NaCl, 2 mmol/L EDTA, 0.5% Triton X‐100, 50 × Roche protease inhibitor) from mice heart or 3dpf zebrafish embryos. The extracts were then fractionated by SDS‐PAGE and transferred to a polyvinylidene difluoride (PVDF) membrane using a transfer apparatus following the manufacturer's instructions (Bio‐rad). Membranes were blocked in 5% non‐fat milk in TBST (10 mmol/L Tris, pH 8.0, 150 mmol/L NaCl, 0.5% Tween 20) for 2 hours followed by TBST wash for 5 minutes. Then membranes were incubated with primary antibodies against NDUFA7 (Abcam, ab140871), GAPDH (Proteintech, 60004‐1‐Ig), and then horseradish peroxidase‐conjugated secondary antibodies as described previously.^16^ All antibodies were diluted in 1:1000 dilution. Membranes were developed with the ECL system (Bio‐rad) following the manufacturer's instructions.

### 2.10 Microscopy

Live embryos were anesthetized using 0.16 mg/mL tricaine methanesulfonate (Sigma) and embedded in 2.5% methyl cellulose (Sigma). Embryos were imaged in bright field and fluorescent mode using fluorescent microscopy (Leica M205 FA). Images were analysed with LAS FA software (Leica) and ImageJ. For myocardial function analysis, embryos were laterally positioned and taken videos at identical levels of magnification and frame rate. Sequential still frames were used to measure ventricular internal chamber dimensions at end diastole (EDD, end‐diastolic diameter) and end systole (ESD, end‐systolic diameter). Fractional shortening (FS) was calculated using the formula (EDD−ESD)/(EDD).

### 2.11 Statistical analysis

Data are presented as mean ± SEM using Image GraphPad Prism 5.0 software (GraphPad Software). Analysis of statistical significance was performed by the Student's *t* test for comparison between two groups. A *P* value of less than .05 was deemed statistically significant.

## 3 RESULTS

### 3.1 The expression level of NDUFA7 is markedly decreased in cardiac hypertrophy

**Figure 1:**
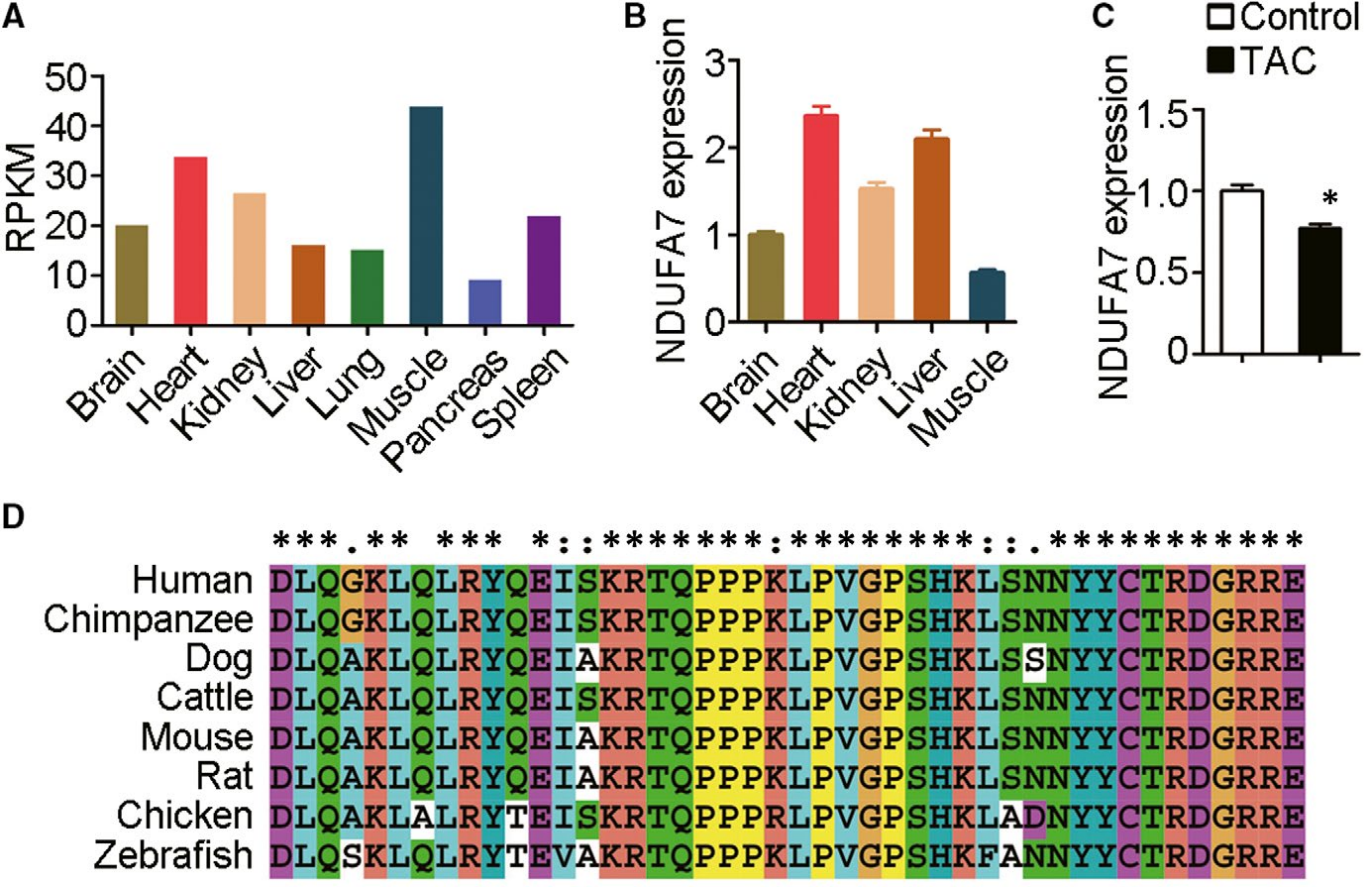
NDUFA7 is involved in cardiac hypertrophy induced by ISO infusion. (A) Tissue expression profiles of NDUFA7 are generated from GTEx database (https://commonfund.nih.gov/gtex). Median RPKM level was shown. (B) RNA was extracted from different tissues of mice, and RT‐qPCR analysis of NDUFA7 and GAPDH expression was then performed. (C) NDUFA7 expression in hearts of mice subjected to cardiac pressure overload by transverse aortic constriction (TAC) or in control group (GSE2459). *P < .05 compared with healthy control. (D) Similarity of Ndufa7 protein sequence from different species including human, chimpanzee, dog, cattle, mouse, rat, chicken and zebrafish

We first checked the expression level of NDUFA7 in tissues using NIH’s Genotype‐Tissue Expression (GTEx) database (https://commonfund.nih.gov/gtex) and found that *NDUFA7* displayed relatively high expression in human heart and muscle (Figure 1A). We extracted RNA from different tissues of mice, performed RT‐qPCR analysis and found high expression level of NDUFA7 mRNA in mice heart, which is consistent with GTEx database finding (Figure 1B). We then searched the GEO (gene expression omnibus) database to explore whether NDUFA7 is involved in cardiac diseases. Compared with control group, NDUFA7 expression was significantly decreased in the mice heart with pressure overload left ventricle hypertrophy caused by transverse aortic constriction (TAC; Figure 1C).^17^ We further analysed the homology of NDUFA7 by aligning the protein sequences from human, chimpanzee, dog, cattle, mouse, rat, chicken as well as zebrafish and found that NDUFA7 is highly conserved among species (Figure 1D). These data indicate that NDUFA7 displays relatively high expression in heart, and its level decreases in cardiac hypertrophy.

### 3.2 Knockdown of *ndufa7* results in cardiac defect in developing zebrafish embryos

**Figure 2:**
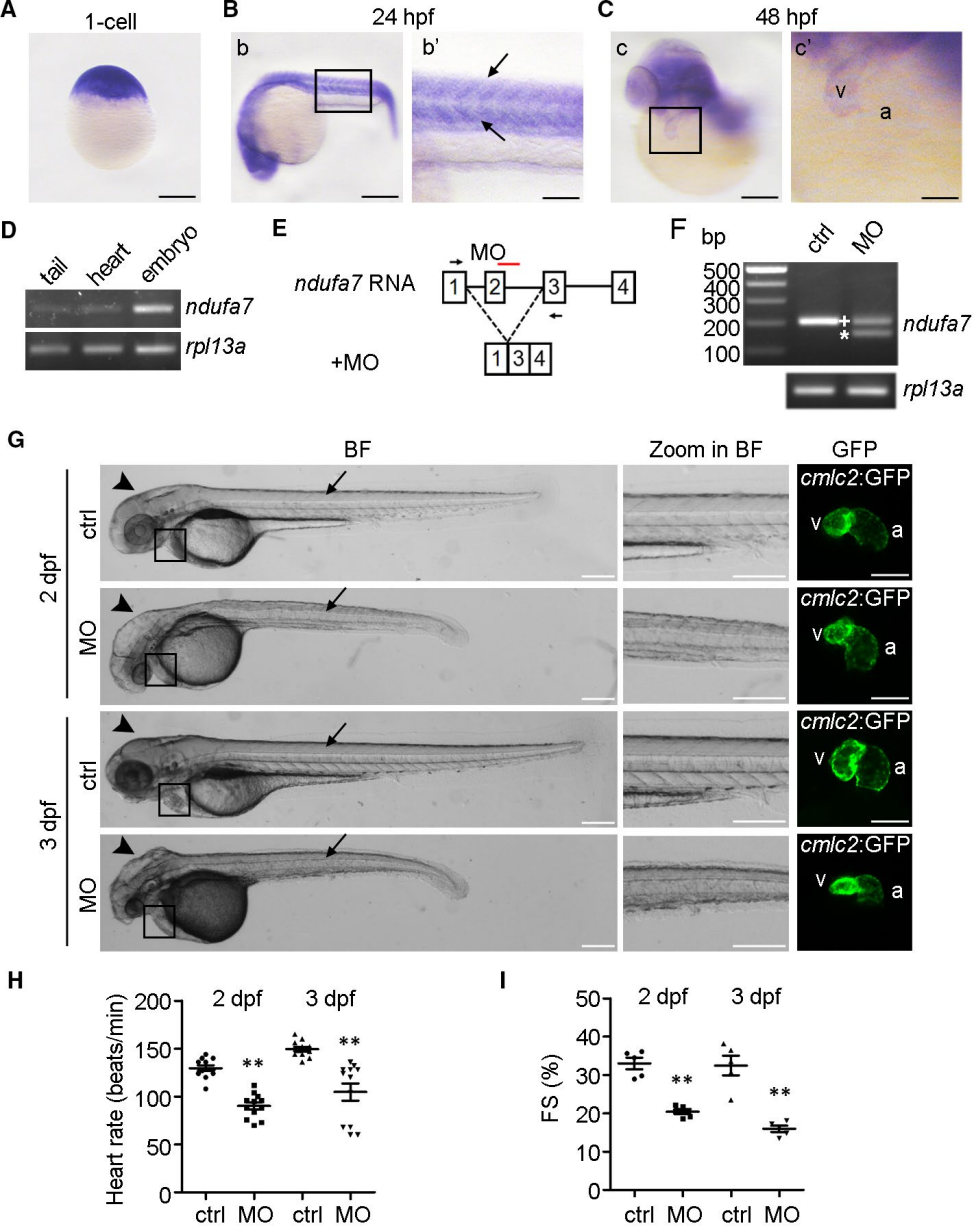
Knockdown of *ndufa7* results in cardiac defect in developing zebrafish embryos. (A‐C) Representative images of whole‐mount in situ hybridization using the *ndufa7* riboprobe were showed. The experiments were performed in triplicate, with 20 embryos per developmental stage. (A) Zebrafish *ndufa7* transcript is broadly expressed at 1‐cell stage. Scale bar, 250 μm. (B) At 24 hpf, *ndufa7* is expressed in the somite (box in b, and arrow in b’). Scale bar, 250 μm in b, 100 μm in b’. (C) By 48 hpf, *ndufa7* is enriched in the heart (box in c), v represents ventricle, and a represents atrium. Scale bar, 250 μm in c, 100 μm in c’. (D) RT‐PCR of *ndufa7* and *rpl13a* RNA isolated from 2 dpf *cmlc2::GFP* zebrafish whole embryo, tail and heart. (E) The *ndufa7* splice morpholino target site and the aberrant transcript. (F) RT‐PCR amplification of RNA from control MO and *ndufa7* MO‐injected zebrafish showing altered splicing of *ndufa7* with either the integration of exon 2 (+) or the partial skipping of exon 2 (*). (G) Morphological defects observed in *ndufa7* morphants (MO) or controls (ctrl) at 2 dpf and 3 dpf. Arrows and arrowheads showed the defects in somites and head, respectively. The fluorescent image demonstrated heart phenotype from the enlarged box region. Scale bar, 300 μm in bright field (BF) and zoom in BF, 150 μm in GFP field. V represents ventricle, and a represents atrium. The experiments were performed in triplicate, processing 40 embryos per condition. (H) WT embryos were injected with MOs at 1‐cell stage, and heart rate was counted via a recorded video captured with the aid of a microscope. Statistical test: Student's t test. **P < .01 compared with controls. n = 12 measurements per condition. (I) Fractional shortening (FS) of the ventricular chamber in control and *ndufa7* morphants was measured at the indicated developmental stages. Statistical test: Student's t test. *P < .05 compared with controls. n = 5 measurements per condition

Given the high homology of *ndufa7* among species, we studied the function of *ndufa7* using zebrafish. To characterize the spatio‐temporal expression pattern of *ndufa7*, we first performed whole‐mount in situ hybridization with different stages of WT zebrafish embryos. *ndufa7* was detected in 1‐cell embryo, suggesting maternal expression of this gene (Figure 2A). We also observed specific somite expression of *ndufa*7 in 24 hpf embryos, and heart expression in 48 hpf embryos (Figure 2B, C). RT‐PCR analysis of RNA isolated from 2 dpf *cmlc2::GFP* zebrafish whole embryo, tail and heart further verified the expression of *ndufa7* in the somite and heart (Figure 2D).

To study the role of *ndufa7* in cardiac development, we employed morpholino (MO)‐based technology to knockdown the *ndufa7* gene in developing zebrafish embryos. Microinjection of splicing MO (e2i2) targeting the splicing donor of exon 2 resulted in aberrant splicing and in turn a frameshift in the resulting ndufa7 protein (Figure 2E). RT‐PCR analysis revealed an extra band resulting from the aberrant splicing (Figure 2F). We then injected control or *ndufa7* MO into 1‐cell *cmlc2::GFP* transgenic zebrafish embryos and found that *ndufa7* morphants displayed a slightly curved, short and roughed tail, as well as a small head at both 2 dpf and 3 dpf stages (Figure 2G). Furthermore, a slight deformed ventricle was observed in 2 dpf embryos with an even more severe heart phenotype presented in 3 dpf embryos. To further investigate the role that *ndufa7* plays in myocardial function, the heart‐beating videos in live embryos were taken. We found that the heart rate of *ndufa7* morphant was markedly reduced compared to that of control group (Figure 2H). Moreover, fractional shortening (FS) measurements showed that *ndufa7* depletion leads to a significant reduction in ventricular function, decreasing from around 34% to 20% at 48 hpf and from approximately 33% to 14% at 72 hpf (Figure 2I). These data demonstrate that *ndufa7* depletion contributes to myocardial dysfunction in zebrafish embryos.

### 3.3 *ndufa7* inhibition contributes to cardiac structural defects

**Figure 3:**
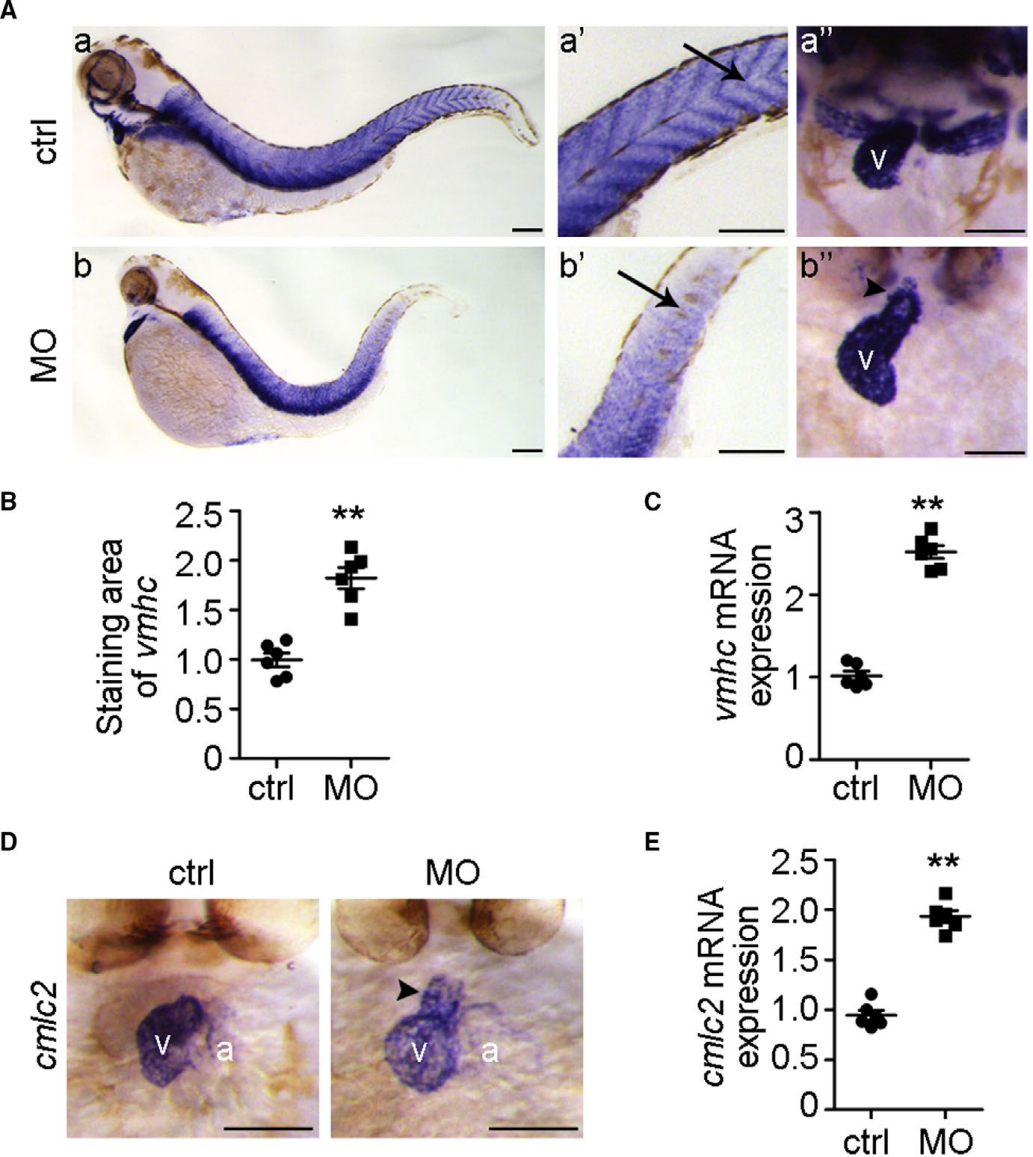
*ndufa7* inhibition contributes to cardiac structural defects. (A) Wild‐type zebrafish embryos were injected with *ndufa7* MO or control MO at 1‐cell stage and then subjected to whole‐mount ISH with riboprobes against *vmhc* at 3 dpf. Lateral views of endogenous *vmhc* mRNA expression pattern were visualized under the microscope (a, b). The expression pattern of *vmhc* mRNA transcripts was expressed in the trunk muscle (a’, b’), as well as in the ventricle under the dorsal views (a’’, b’’). Arrows and arrowheads indicate the muscle structure and outflow track, respectively, and v represents ventricle. Scale bar, 75 μm. The experiments were performed in triplicate, with 20 individuals per condition. (B) Experiments were performed as in (A), and the staining area of vmhc in the heart was examined using ImageJ software. Statistical test: Student's t test. **P < .01 compared with control MO group, n = 6 measurements per condition. (C) *cmlc2::GFP* embryos were injected with *ndufa7* MO or control MO at 1‐cell stage, and RNA was then extracted from harvested embryo hearts at 3 dpf. The expression level of vmhc in the hearts was examined by RT‐qPCR. Statistical test: Student's t test. **P < .01 compared with control MO group, n = 6 measurements per condition. (D) Wild‐type zebrafish embryos were injected with *ndufa7* MO or control MO at 1‐cell stage, and then subjected to whole‐mount ISH with riboprobes against *cmlc2* at 3 dpf. Dorsal views of endogenous *cmlc2* mRNA expression pattern were observed. Arrowheads indicate the outflow track, v represents ventricle and a represents atrium. Scale bar, 75 μm. The experiments were performed in triplicate, with 20 individuals per condition. (E) *cmlc2::GFP* embryos were injected with *ndufa7* MO or control MO at 1‐cell stage, and RNA was then extracted from embryo hearts at 3 dpf for RT‐qPCR. Statistical test: Student's t test. *P < .05 compared with control MO group, n = 6 measurements per condition

To investigate whether the *ndufa7* MO‐induced cardiac dysfunction is due to the structure defect, we evaluated the cardiac phenotype with heart‐chamber marker *vmhc* (*ventricular myosin heavy chain*). Whole‐mount ISH performed on 3 dpf zebrafish embryos showed that vmhc mRNA is specifically expressed in the skeletal muscle of the trunk as well as in cardiac ventricular muscle (Figure 3A). Compared with the control group, *ndufa7* morphants exhibited a small head and disorganized somite structure missing the paralleled V shape (Figure 3A‐a,b,a’,b’). The dorsal view of the embryo hearts showed that *ndufa7* morphants exhibit an enlarged ventricle and wide outflow track (Figure 3A‐a",b"). The size of the ventricle in *ndufa7* morphant group was around 1.9 times that of the control group (Figure 3B). We then injected *cmlc2::GFP* embryos with *ndufa7* MO or control MO and extracted RNA from collected embryo hearts at 3 dpf. The qPCR analysis showed that depletion of *ndufa7* significantly increases the expression level of vmhc in the heart (Figure 3C).

To verify the above result, we further performed whole‐mount ISH with another heart‐chamber marker *cmlc2* (*cardiac myosin light chain 2*). cmlc2 was shown to be predominantly expressed in the ventricle and was slightly expressed in the outcurvature of the atrium (Figure 3D). Similarly, compared with the control group, *ndufa7* morphants displayed a swelling ventricle with wide outcurvature, as well as elongated outflow track. Furthermore, the expression level of *cmlc2* in the heart was significantly upregulated upon *ndufa7* knockdown as measure by qPCR (Figure 3E). These data show that inhibition of *ndufa7* leads to altered cardiac structure.

### 3.4 The cardiac hypertrophy biomarkers *nppb* and *nppa* are upregulated by *ndufa7* depletion

**Figure 4:**
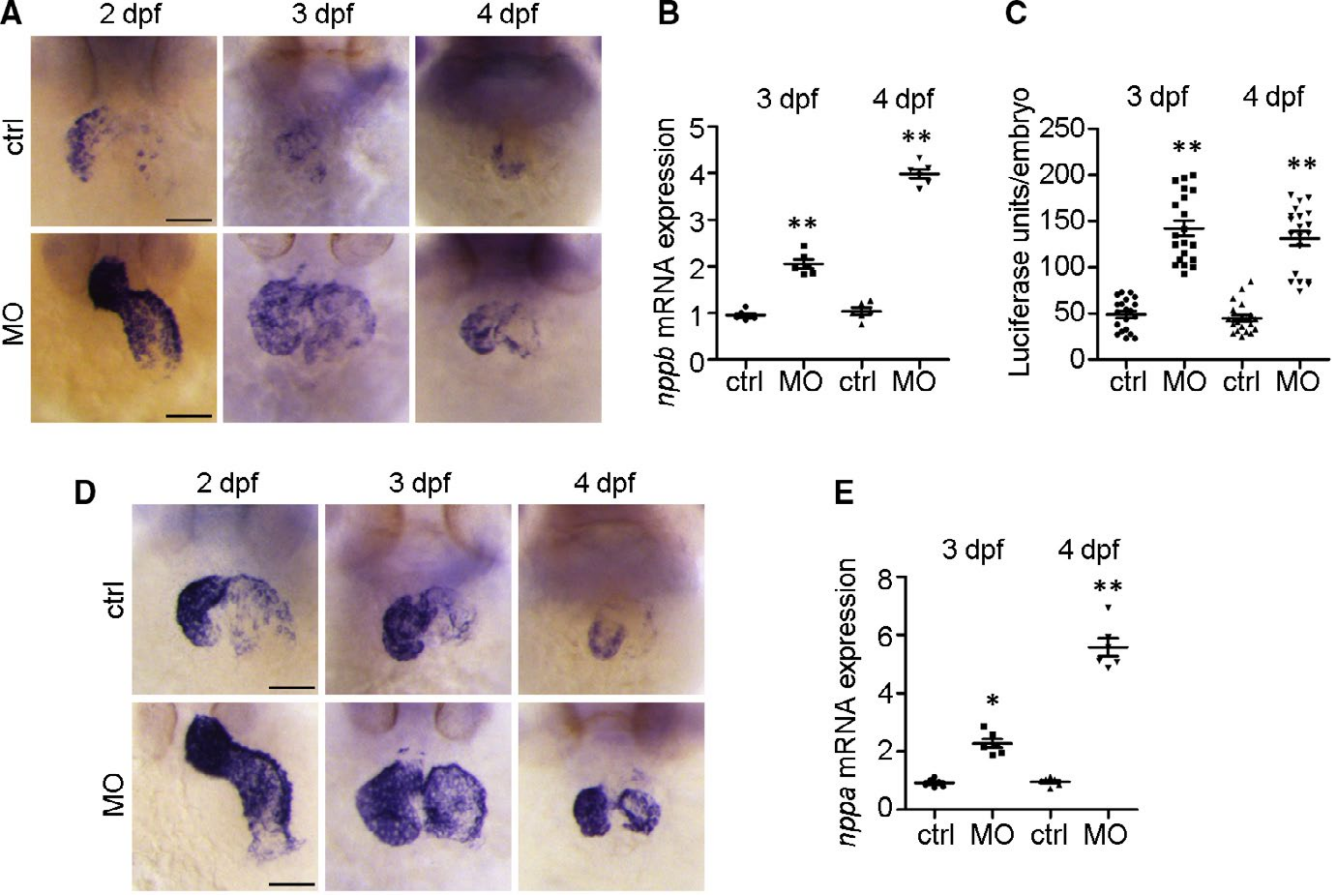
The cardiac hypertrophy biomarkers *nppb* and *nppa* are upregulated by *ndufa7* depletion. (A) WT zebrafish embryos were injected with *ndufa7* MO or control MO and then subjected to whole‐mount in situ hybridization with riboprobes against *nppb*. Scale bar, 75 μm. The experiments were performed in triplicate, with 20 individuals per condition. (B) *cmlc2::GFP* embryos were injected with *ndufa7* MO or control MO at 1‐cell stage, and RNA was then extracted from harvested embryo hearts at 3 dpf or 4 dpf for RT‐qPCR. The experiments were performed in triplicate, with 60 individuals per condition. Statistical test: Student's *t* test. **P < .01 compared with control MO group, n = 6 measurements per condition. (C) *nppb::F‐luc* embryos were injected with *ndufa7* splice‐blocking MO or control MO at 1‐cell stage and collected at 3 dpf and 4 dpf for the luciferase assay. The experiments were performed in triplicate, with n = 30 individuals per condition. Statistical test: Student's t test. **P < .01 compared with control MO group, n = 20 measurements per condition. (D) Wild‐type zebrafish embryos were injected with *ndufa7* MO or control MO and then subjected to whole‐mount in situ hybridization with riboprobes against *nppa* probe. Scale bar, 75 μm. The experiments were performed in triplicate, with 20 individuals per condition. (E) *cmlc2::GFP* embryos were injected with *ndufa7* MO or control MO at 1‐cell stage, and RNA was then extracted from harvested embryo hearts at 3 dpf or 4 dpf for RT‐qPCR. The experiments were performed in triplicate, with 60 individuals per condition. Statistical test: Student's t test. **P < .01, *P < .05 compared with control MO group, n = 6 measurements per condition

To examine whether natriuretic peptide signalling is implicated in *ndufa7*‐MO‐induced cardiac hypertrophy, we first performed whole‐mount ISH to examine the alteration of *nppb* upon *ndufa7* depletion. As shown in Figure 4A, *nppb* was mainly expressed in ventricle, with its expression level decreased during the development in the control group. In contrast, the *nppb* expression level in *ndufa7* morphants remained high and elevated during the development. In particular, the ventricle chamber of *ndufa7* morphant was enlarged compared to the control group at 3 dpf. Moreover, the qPCR analysis showed that *ndufa7* morphants exhibited a significantly higher *nppb* mRNA expression, with an average 1‐fold increase at 3 dpf and 2.8‐fold increase at 4 dpf (Figure 4B). We then performed a luciferase assay with *nppb::F‐Luc* transgenic zebrafish line, a genetic model to study HCM signalling. In comparison with the control group, depletion of *ndufa7* significantly increased the *nppb* promoter activity, increasing luciferase activity by around 1.5‐fold (Figure 4C).

To further confirm the role of *ndufa7* in cardiac hypertrophy, we performed whole‐mount ISH with another hypertrophic marker *nppa*. It has been shown that *nppa* is expressed both in ventricle and atrium. Similar to *nppb*, its expression level decreased during development in the control group and the expression level of *nppa* in *ndufa7* morphants was increased compared with control group (Figure 4D). Interestingly, the staining pattern of *nppa* at 3 dpf larval zebrafish also indicated that the ventricle chamber of *ndufa7* morphant is enlarged as compared with control. We also showed that knockdown of *ndufa7* enhances expression level of *nppa* in the heart by qPCR analysis, with an average 1.4‐fold increase at 3 dpf and 5.6‐fold increase at 4 dpf (Figure 4E). These results show that *ndufa7* depletion contributes to increased expression of pathological hypertrophy markers.

### 3.5 Calcineurin signalling is involved in *ndufa7* inhibition induced cardiac hypertrophy

**Figure 5:**
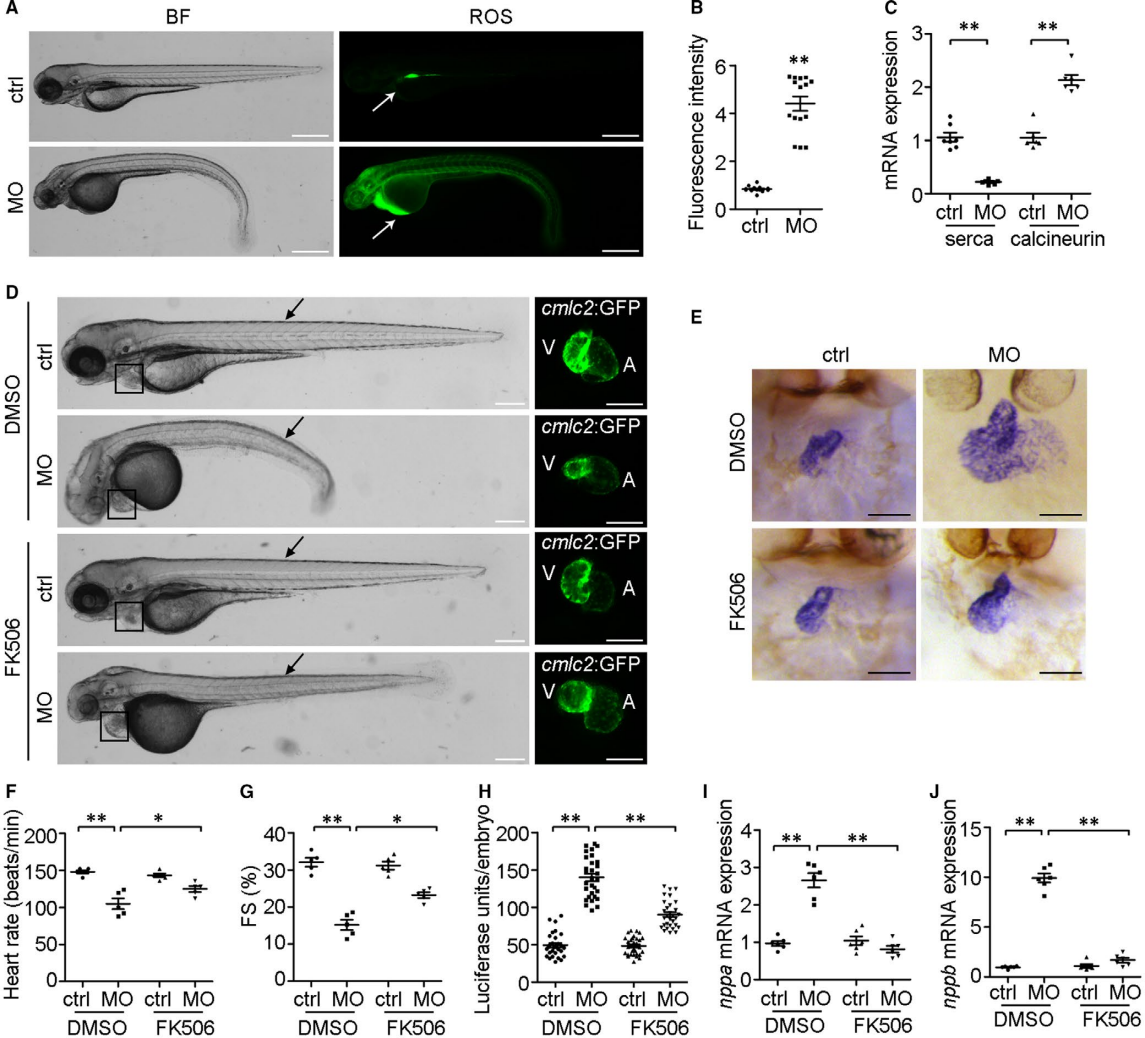
Calcium signalling is involved in *ndufa7*‐depletion induced cardiac hypertrophy. (A) WT zebrafish embryos were injected with MO at 1‐cell stage and stained for ROS using 2’,7’‐dichlorofluorescin diacetate. Arrows indicate the ROS staining in the heart. Scale bar, 500 μm. (B) Experiments were performed as in (A), and the intensity of ROS level was analysed. **P < .01 compared with control MO group. (C) *cmlc2::GFP* embryos were injected with *ndufa7* MO or control MO at 1‐cell stage, and RNA was then extracted from harvested embryo hearts at 3 dpf for RT‐qPCR. The expression level of serca and calcineurin were examined. The experiments were performed in triplicate, with 60 individuals per condition. Statistical test: Student's t test. **P < .01 compared with control MO group. (D) MO microinjections were performed at 1‐cell stage, 0.5% DMSO (vehicle control) or FK506 (in 0.5% DMSO) were added to embryo water at 21 hpf and cardiac fluorescent imaging was performed on *cmlc2::GFP* embryos at 3 dpf. Black arrow indicates somite defects, and black box shows cardiac defects. Scale bar, 300 μm in bright field, 150 μm in GFP field. The experiments were performed in triplicate, processing 30 embryos per condition. (E) Wild‐type zebrafish embryos were injected with MO at 1‐cell stage, treated with DMSO or FK506 at 21 hpf, and then subjected to whole‐mount ISH with riboprobes against *cmlc2* at 3 dpf. Dorsal views of endogenous *cmlc2* mRNA expression pattern were observed. Scale bar, 75 μm. (F‐G) Experiments were performed as in (D), heart rate and fractional shortening (FS) of the ventricular chamber were measured. **P < .01, *P < .05 compared with controls. (H) *nppb::F‐luc* embryos were injected with *ndufa7* splice‐blocking MO or control MO at 1‐cell stage, treated with FK506 or 0.5% DMSO at 21 hpf, and collected at 3 dpf for the luciferase assay. The experiments were performed in triplicate, with 50 individuals per condition. Statistical test: Student's *t* test. **P < .01 compared with control MO group, n = 30 measurements per condition. (I‐J) *cmlc2::GFP* embryos were injected with *ndufa7* MO or control MO at 1‐cell stage, and treated with 0.5% DMSO or FK506. RNA was extracted from harvested embryo hearts at 3 dpf to examine the mRNA expression level of *nppa* and *nppb*. The experiments were performed in triplicate, with 60 individuals per condition. Statistical test: Student's *t* test. **P < .01 compared with control MO group or DMSO vehicle group, n = 6 measurements per condition

To gain mechanistic insight into the role of *ndufa7* in cardiac hypertrophy, we first examined the impact of *ndufa7* deficiency on ROS level by staining embryos with 2’,7’‐dichlorofluorescin diacetate. A significant increase in ROS level especially in zebrafish heart was observed in *ndufa7* morphant compared with control group (Figure 5A, B). It has been reported that calcineurin signalling regulates cardiac hypertrophy with accompanying by the intracellular ROS production.^18^ We further examined several important genes in calcineurin signalling and found that *ndufa7* MO significantly reduces serca2 level and increase calcineurin level in embryonic hearts (Figure 5C).

To further verify our hypothesis, we treated *ndufa7*‐MO‐injected embryos with the calcineurin inhibitor, FK506. In the vehicle (DMSO) treated group, *ndufa7* morphant presented a curved and roughed tail and evidently defected ventricle (Figure 5D). However, *ndufa7* morphant treated with FK506 exhibited a straight tail and less defected ventricle. Besides, whole‐mount ISH analysis with cmlc2 showed that FK506 treatment restored *ndufa7* depletion induced swelling ventricle and wide outcurvature to an extent (Figure 5E). Moreover, FK506 treatment partially rescued defects in heart rate and ventricle function in *ndufa7* morphant (Figure 5F, G). To further investigate whether calcineurin signalling is involved in *ndufa7* inhibition induced cardiac hypertrophy, we performed a luciferase assay with *nppb:Luc* zebrafish embryos treated with FK506. As shown in Figure 5H, FK506 treatment restored the *ndufa7* MO‐induced upregulation of nppb. Moreover, we found that inhibition of calcineurin restored the expression level of both nppa and nppb in embryo hearts by qPCR analysis (Figure 5I, J). These results show that calcineurin signalling is involved in *ndufa7* induced cardiac hypertrophy.

### 3.6 Depletion of NDUFA7 leads to cardiac cell hypertrophy

**Figure 6:**
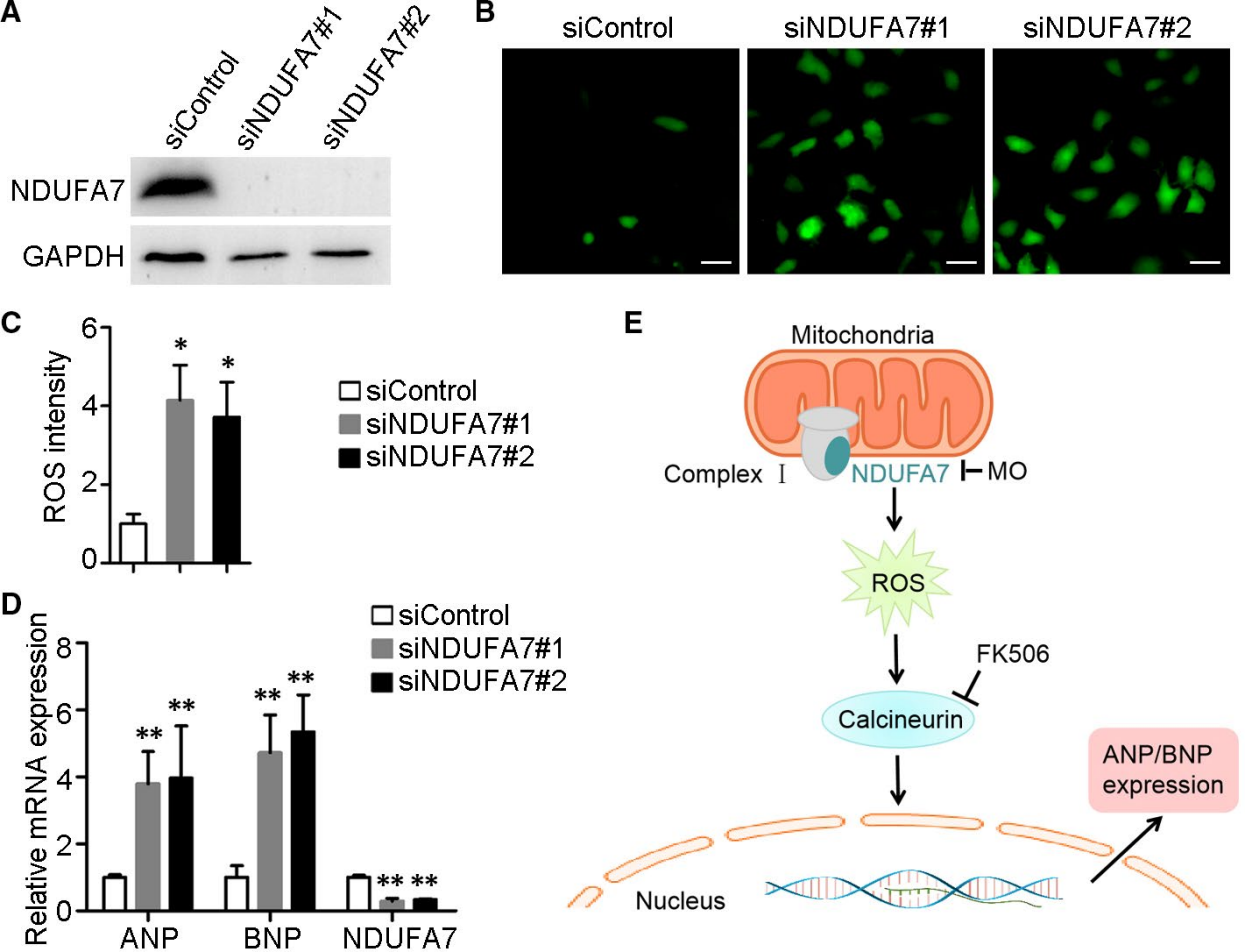
Depletion of NDUFA7 leads to cardiac hypertrophy in H9c2 cells. (A) H9c2 cells were transfected with control or NDUFA7 siRNAs for 72 hours, and expression level of NDUFA7 and GAPDH were examined. (B) H9c2 cells transfected with control or NDUFA7 siRNAs were stained for ROS using 2’,7’‐dichlorofluorescin diacetate, and then visualized under the fluorescence microscope. Scale bar, 50 µm. (C) Experiments were performed as in (B), and the ROS intensity in each group were examined by ImageJ. *P < .05 compared with control group. (D) H9c2 cells transfected with control or NDUFA7 siRNAs for 72 h, RNA was then extracted for RT‐qPCR analysis. **P < .01 compared with control group. (E) Schematic representation of the proposed mechanism. Depletion of *ndufa7* triggered ROS production and subsequent calcineurin signalling activation, which further led to the expression of cardiac hypertrophy genes

To verify the above findings in zebrafish, we further examined the role of NDUFA7 in cardiac cells. H9c2 cells, derived from embryonic rat heart tissue, have been used as a cardiac cell model in many cardiac hypertrophy studies.^19^, ^20^ Immunoblotting revealed that NDUFA7 siRNAs decreased the protein level of NDUFA7 effectively (Figure 6A). Moreover, NDUFA7 depletion promotes ROS generation, which is consistent with the phenotype in zebrafish heart (Figure 6B, C). We further examined expression of cardiac hypertrophy markers by qPCR analysis and found that NDUFA7 inhibition markedly increase the mRNA level of ANP and BNP, indicating that depletion of NDUFA7 leads to cardiac hypertrophy (Figure 6D). We proposed that depletion of *ndufa7* promoted ROS production and calcineurin signalling activation, leading to the expression of cardiac hypertrophy genes, thus contributing to cardiac hypertrophy (Figure 6E).

## 4 DISCUSSION

Due to the embryo transparency and the ease of direct embryonic manipulation, the zebrafish has emerged as a promising research tool to model cardiovascular diseases including cardiac hypertrophy, congenital heart defects, arrhythmia, as well as cardiomyopathy. For example, a zebrafish model of human cardiac troponin T (TNNT2) mutation known to cause HCM has been generated.^21^ The morphant zebrafish embryos with *tnnt2* knockdown displayed sarcomere disarray and a robust induction of myocardial hypertrophic pathways, which is similar to humans with HCM.^22^ Moreover, zebrafish with *pickwick* ^m171^ mutation demonstrated reduced cardiac contractile function and pericardial oedema.^23^ The mutation was attributed to the gene *ttn*, encoding the protein Titin, which is an important cause of human idiopathic dilated cardiomyopathy.^24^ Besides, the establishment of zebrafish models to dissect ANP/BNP signalling pathway further prompts the functional studies of cardiac hypertrophy pathogenesis, disease mechanisms and potentially drug screens.^25^, ^26^, ^27^

*NDUFA7* has been reported to be associated with rheumatoid arthritis (RA) with severe erosive arthritis.^12^ Till now, the biological functions of *NDUFA7* remain unknown. In this study, we employed zebrafish models extensively to explore *ndufa7* function in pathological cardiac hypertrophy. Consistent with tissue expression database, zebrafish *ndufa7* is expressed in the heart and muscle during embryonic development. We further provide several lines of evidence showing the important role of *ndufa7* in cardiac hypertrophy: Knockdown of *ndufa7* leads to cardiac defect in developing zebrafish embryos; *ndufa7* depletion contributes to the elevated expression of hypertrophic biomarkers *nppb* and *nppa*; calcineurin signalling is involved in *ndufa7* inhibition induced cardiac hypertrophy. These findings were further verified in the model of cardiac cells. It is possible that *ndufa7* depletion activates calcineurin signalling, which allows the dephosphorylated NFAT to be imported into the nucleus, thus leading to the expression of cardiac hypertrophy genes. It will be interesting to explore in further studies, which will deepen our understanding of the biological function of *ndufa7*. Given the high density of mitochondria in cardiomyocytes, it is not surprising that *ndufa7* plays an important role in cardiac function.^28^

Mitochondrial function is essential for proper heart function by producing a constant supply of energy to accomplish complex cellular processes including continuous repetitive contraction and maintenance of Ca^2+^ homeostasis.^29^ Oxidative stress, characterized by the accumulation of ROS, is known to exert detrimental influence on the myocardium such as the induction of apoptotic cell death, hypertrophy and dysfunction. Increasing evidence suggests that cardiac hypertrophy induced by mechanical left ventricular wall stress is partially triggered by ROS generation.^30^, ^31^ It has been reported that calcineurin regulates the pathogenesis of cardiac hypertrophy with accompanying by the intracellular ROS production.^10^ Mitochondrial respiratory complex I has been considered to be the major ROS generation site because large changes in the potential energy of the electrons occur in the sites.^32^ In a recent study, it is indicated that ROS might involve in the NDUFA7 induced rheumatoid arthritis.^12^ In this study, we demonstrate that depletion of *ndufa7* promotes ROS production and calcineurin signalling activation, leading to cardiac hypertrophy. Furthermore, alterations in mitochondrial metabolism have been reported in pathological cardiac hypertrophy, including dysfunction of the electron transport chain and reduced capacity of ATP synthesis.^33^ It is possible that *ndufa7* might be associated with cardiac hypertrophy by regulating the activity of mitochondria complex I, as well as the ATP generation of mitochondrial oxidative phosphorylation. Elucidation of this will help to fully understand the role of *ndufa7* in the pathogenesis of cardiac hypertrophy.

## CONFLICT OF INTEREST

The authors declare that they have no competing interests.

## AUTHOR CONTRIBUTION

**Xingjuan Shi:** Conceptualization (lead); Data curation (lead); Formal analysis (lead); Funding acquisition (lead); Investigation (lead); Methodology (lead); Supervision (lead); Validation (lead); Visualization (lead); Writing‐original draft (lead); Writing‐review & editing (lead). **Yu Zhang:** Data curation (supporting); Investigation (supporting). **Ru Chen:** Data curation (supporting); Investigation (supporting); Methodology (supporting). **Yijie Gong:** Data curation (supporting); Investigation (supporting); Methodology (supporting). **Mingming Zhang:** Methodology (supporting). **Rui Guan:** Validation (supporting); Visualization (supporting). **Ori D. Rotstein:** Resources (supporting). **Xiangdong Liu:** Funding acquisition (supporting); Resources (supporting); Supervision (supporting). **Xiao‐Yan Wen:** Conceptualization (supporting); Funding acquisition (supporting); Resources (lead).

## Data Availability

The data that support the findings of this study are available on request from the corresponding author. The data are not publicly available due to privacy or ethical restrictions.
